# Supplementary material for: Synthesis, Self-Assembly and Characterization of Tandem Triblock BPOSS-PDI-X Shape Amphiphiles
Source: Molecules. 2019 Jun 4;24(11):2114. doi: 10.3390/molecules24112114 (PMC6600600; doi:10.3390/molecules24112114)
Supplement: Supplementary file 1 [file molecules-24-02114-s001.pdf]

molecules MDPI

Supporting Information

Synthesis, Self-Assembly and Characterization of Tandem Triblock BPOSS-PDI-X Shape Amphiphiles

Yu Shao,[1,†] Jia Chen,[1,†] Xiang-Kui Ren,[2] Xinlin Zhang,[1] Guang-Zhong,[3] YinXiaopeng Li,[4] Jing Wang,[5] Chrys Wesdemiotis,[6] Wen-Bin Zhang,[3] Shuguang Yang,[1,*] Bin Sun,[1,*] and Meifang Zhu[1,*]

1 State Key Laboratory for Modification of Chemical Fibers and Polymer Materials and College of Material Science and Engineering, Center for Advanced Low-Dimension Materials, Donghua University, Shanghai 201620, China
2 School of Chemical Engineering and Technology, Tianjin University, Tianjin 300350, China
3 Key Laboratory of Polymer Chemistry and Physics of Ministry of Education, Center for Soft Matter Science and Engineering, College of Chemistry and Molecular Engineering, Peking University Beijing 100871, P. R. China
4 Department of Chemistry, University of South Florida, 4202 East Fowler Ave, Tampa, Florida 33620, USA
5 South China Advanced Institute of Soft Matter Science and Technology, South China University of Technology, Guangzhou 510640, P. R. China
6 Department of Chemistry, The University of Akron, Akron, OH 44325, USA
* Correspondence: shgyang@dhu.edu.cn (S. Yang); sunbin@dhu.edu.cn (B. Sun); zmf@dhu.edu.cn (M. Zhu)
† These authors have contributed equally to the work.

Experimental Section

Single Crystal Growth. The single crystals of BPOSS-PDI-OH were obtained by slow evaporation from dilute solution on substrates in a solvent-saturated atmosphere. A chamber with a THF saturated atmosphere was constructed using a culture dish with a steel cylindrical mount at the bottom and 1 ml of THF at the bottom of the dish. A square substrate of about 1.0 × 1.0 cm^2 (e.g. carbon-coated mica) was placed on the top of the mount and the dish was sealed with a glass cover. The sealed chamber was kept at room temperature for 1h for the atmosphere to reach THF saturation. The solvent was allowed to slowly evaporate through the interface between the glass cover and the dish wall. A micro-syringe was used to deposit one drop of the dilute solution on the substrate. The chamber was then left to allow for the solvent to completely evaporate. The single crystals on the substrate were examined by optical microscopy (Olympus BX52) and then collected for characterization using a variety of techniques.

Instrumentations. The NMR spectra were measured on a Varian 500 NMR spectrometer and Varian Mercury 300 with chloroform ($CDCl_3$, Aldrich, ⩾99.8) as the solvent. The ^1H NMR spectra were referenced to the residual proton impurities in the $CDCl_3$ at δ 7.27 ppm; the ^{13}C NMR spectra were referenced to $CDCl_3$ at δ 77.00 ppm. Infrared spectra were recorded on an Excalibur Series FT-IR spectrometer (DIGILAB, Randolph, MA) by casting polymer films on KBr plates from polymer solutions with subsequent drying. The data were processed using Win-IR software. Matrix-assisted laser desorption/ionization time-of-flight (MALDI-TOF) mass spectra were recorded on a Bruker Reflex-III TOF/TOF mass spectrometer (Bruker Daltonics, Billerica, MA), equipped with a Nd:YAG laser emitting at a wavelength of 355 nm with *trans*-2-[3-(4-tertbutylphenyl)-2-methyl-2-propenylidene] malononitrile (DCTB, Aldrich, >99%) as the matrix in $CHCl_3$ at a concentration of 20 mg mL^{-1}.

Differential scanning calorimeter (DSC, Perkin-Elmer PYRIS Diamond with Intracooler 2P cooling system) experiments were carried out to observe possible phase transitions. The samples were grown as stated above and collected in a DSC pan with a weight of typically 3 mg. The samples were then heated at a rate of 10 °C min^{-1} from −25 °C to 200 °C. The thermogravimetric analysis (TGA) of the samples was analyzed with a TA Instrument-Water LLC Q500. The samples were grown as

stated above and collected with a weight of typically 3 mg. The samples were heated at a rate of 10 °C min^{-1} from 0 °C to 800 °C under the protection of nitrogen (40 mL/min).

One-dimensional (1D) wide angle X-ray diffraction (WAXD) experiments were conducted using a Rigaku Multiflex 2 kW automated diffractometer using Cu Kα radiation (0.1542 nm) in reflection mode. The detector scanning rate was 1° per minute, and the 2θ angle was ranged between 1.7° and 20.0°. The peak positions were calibrated using silicon powder in the high angle region (>15°) and silver behenate in the low angle region (<15°). Background scattering was subtracted from the sample patterns. In order to observe different types of crystal, various solution evaporation rates were applied (from slow to very slow) to form single crystal mats either directly on the X-ray sample holder or in a well-sealed vial before being deposited onto the X-ray sample holder.

For transmission electron microscopy (TEM, Philips Tecnai 12 at an accelerating voltage of 120 kV) experiments, the single crystals were prepared on a carbon coated mica surface. Clean TEM copper grids (400 mesh, SPI) were then used to pick up the film. Before TEM observation, samples were put into a vacuum oven for 12 hours to remove the residual solvent and moisture. Bright field (BF) images and selective area electron diffraction (SAED) patterns were then taken. The d-spacings were calibrated using a TiCl standard.

UV/Vis spectra were recorded on a Lambda 35 (Perkin Elmer) spectrophotometer using 1 mm cuvette. The excitation and emission spectra of the samples were investigated on a FP-6600 steady-state fluorescence spectrophotometer. Sample solutions with different concentrations are put still overnight to reach the equilibrium state and confirmed no precipitates before testing. All the measurements were carried out at room temperature in ambient air and the analysis range was 200-800 nm with a resolution of 1nm.

Electrospray ionization (ESI) mass spectra were obtained on a Waters Synapt HDMS quadrupole/time-of-flight (Q/ToF) tandem mass spectrometer equipped with traveling wave ion mobility (TWIM) separation. The TWIM device is located between the Q/ToF mass analyzers and consists of three parts, a trap cell, ion mobility (IM) cell, and a transfer cell. The trap and transfer cells can be used for conventional tandem mass spectrometry experiments via collisionally activated dissociation (CAD). The ion mobility cell is used in IM separations. The following parameters were used in the TWIM-MS experiments: ESI capillary voltage, 3.5 kV; sample cone voltage, 35 V; extraction cone voltage, 3.2 V; desolvation gas flow, 800 L/h (N_2); trap collision energy (CE), 6 eV; transfer CE, 4 eV; trap gas flow, 1.5 mL/min (Ar); IM gas flow, 22.7 mL/min (N_2); sample flow rate, 10 μL/min; source temperature, 30 °C; desolvation temperature, 40 °C; IM traveling wave velocity, 380 m/s; and IM traveling wave height, 15 V. In the tandem mass spectrometry (MS/MS) experiments, argon was used as the collision gas and the trap CE was varied from 6 to 37 eV to cause fragmentation. The sprayed solution was prepared by dissolving ~1 mg of sample in 1 mL of a MeOH/THF (*v*/*v*) 1/3 mixture and adding 5 μL of 10 mg/mL NaTFA in THF as the cationizing agent.

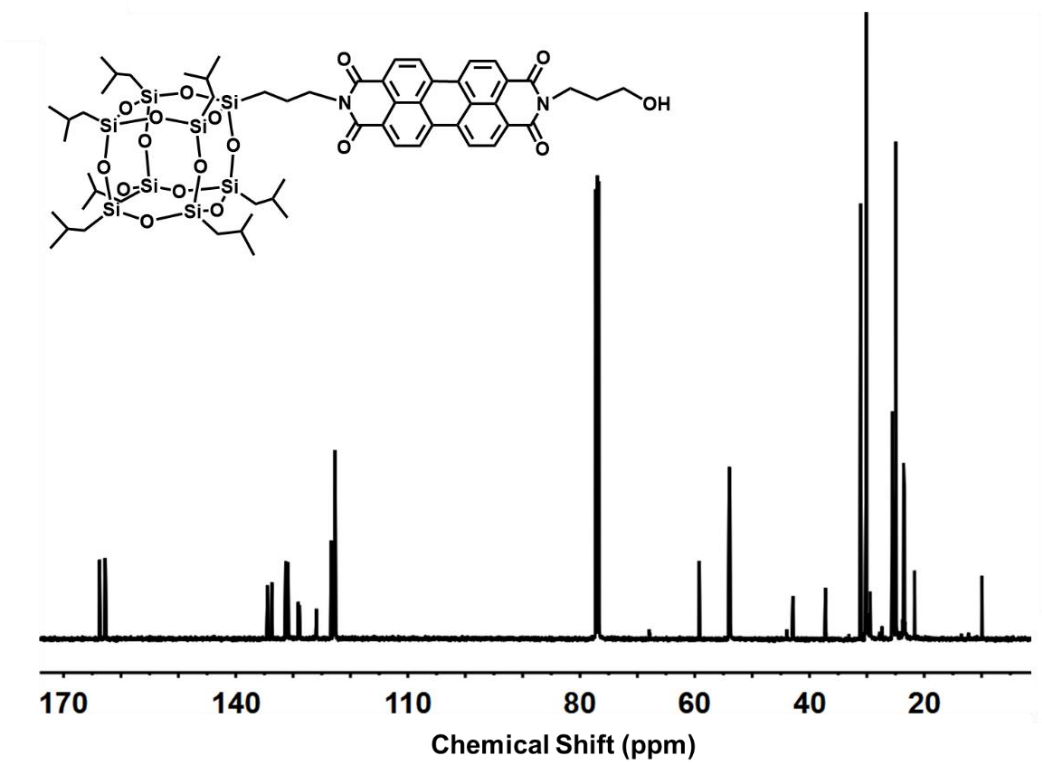

Figure S1. ^{13}C NMR spectrum of BPOSS-PDI-OH.

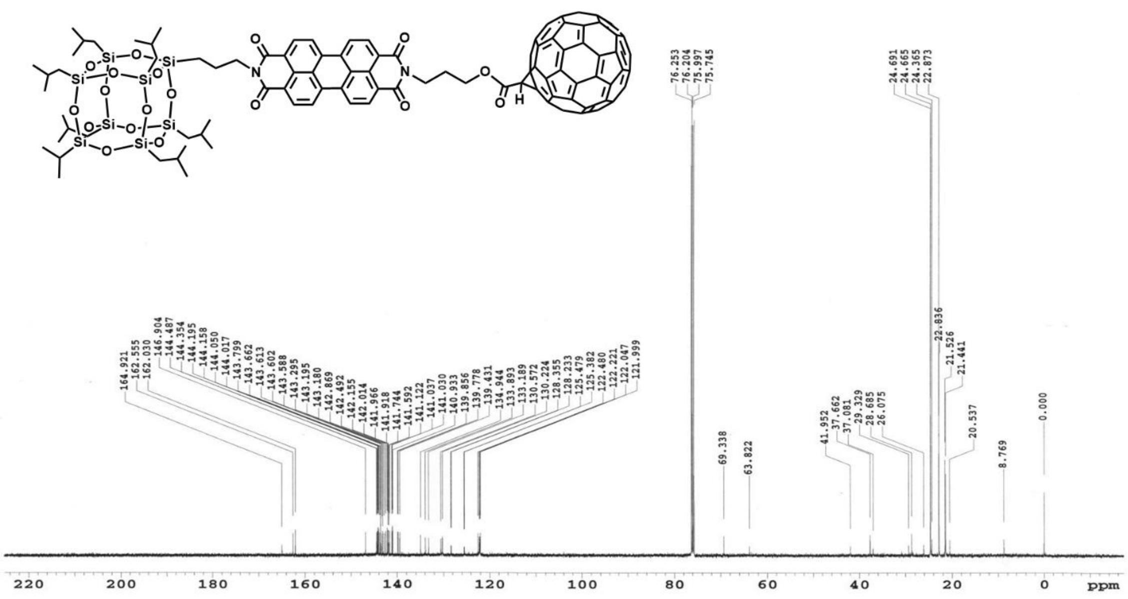

Figure S2. ^{13}C NMR spectrum of BPOSS-PDI-C_{60}.

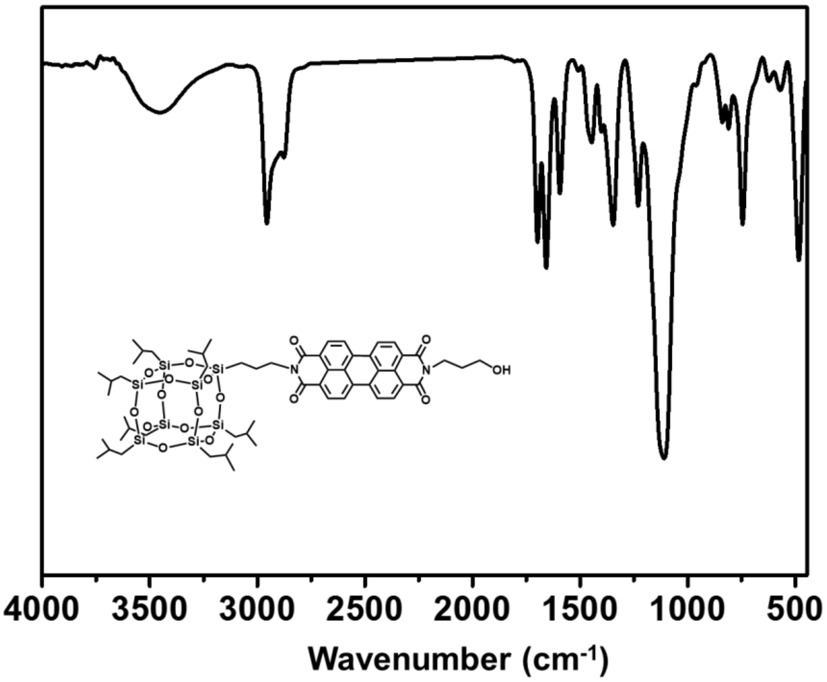

Figure S3. FT-IR spectrum of BPOSS-PDI-OH.

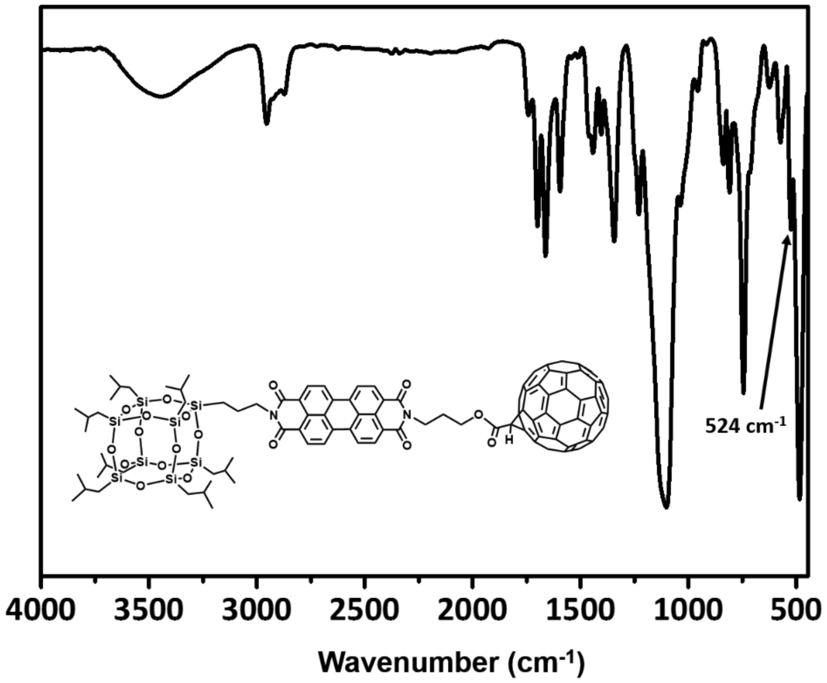

Figure S4. FT-IR spectrum of BPOSS-PDI-C_{60}.

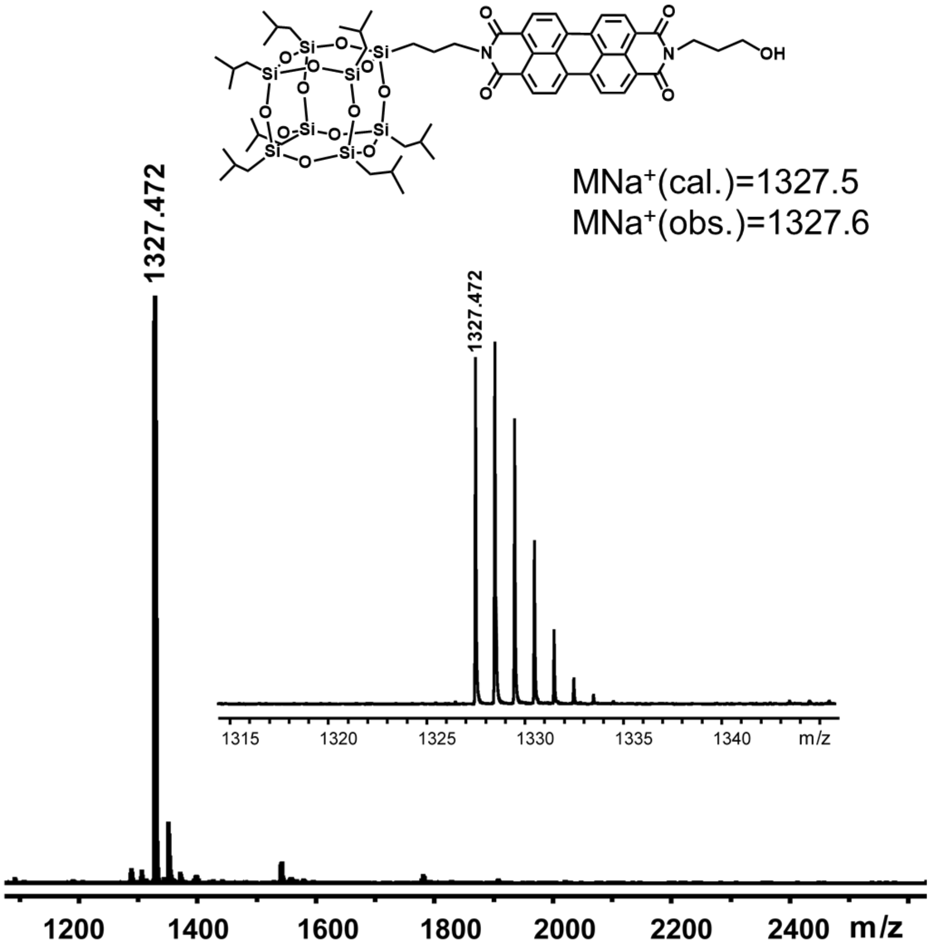

Figure S5. MALDI-TOF MS spectrum of BPOSS-PDI-OH.

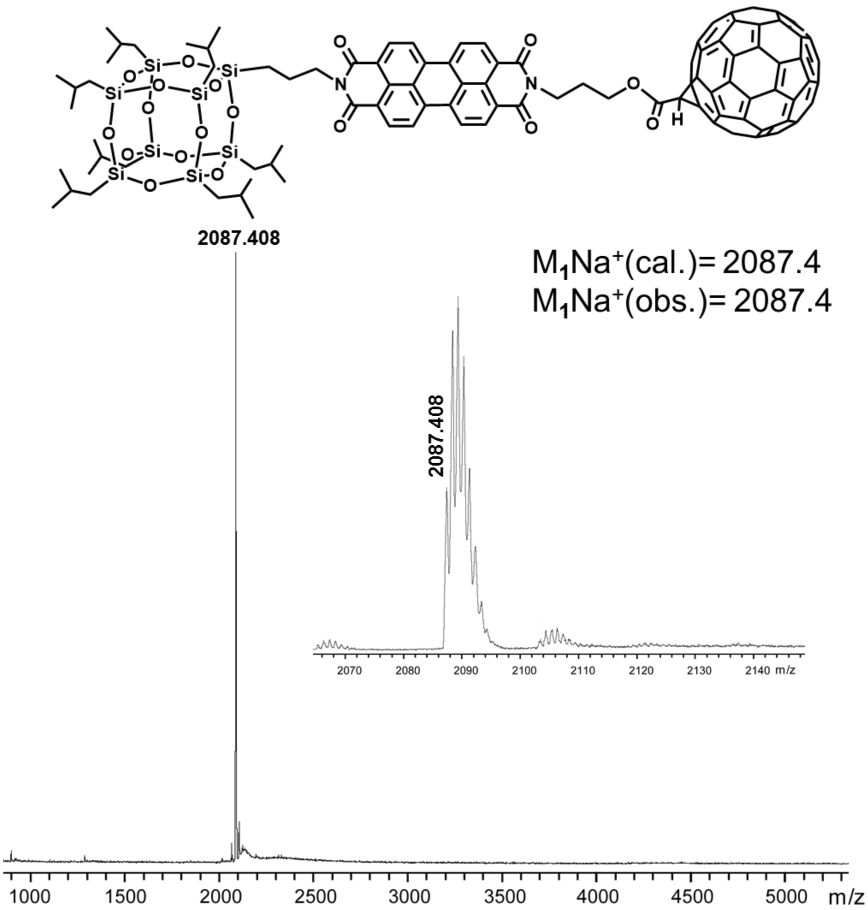

Figure S6. MALDI-TOF MS spectrum of BPOSS-PDI-C_{60}.

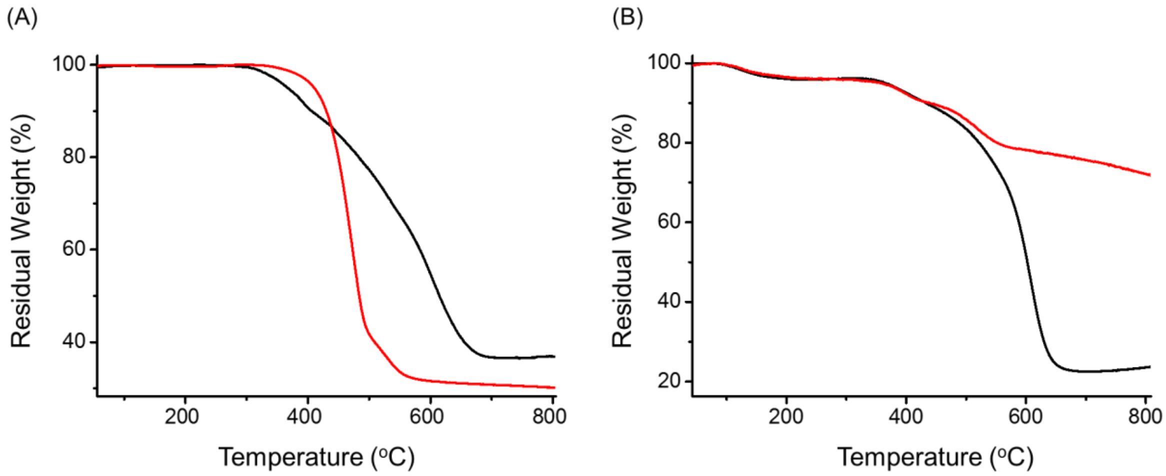

Figure S7. TGA thermograms of (**A**) BPOSS-PDI-OH and (**B**) BPOSS-PDI-C_{60}. The red line is that measured under nitrogen and the black line is that measured under air.

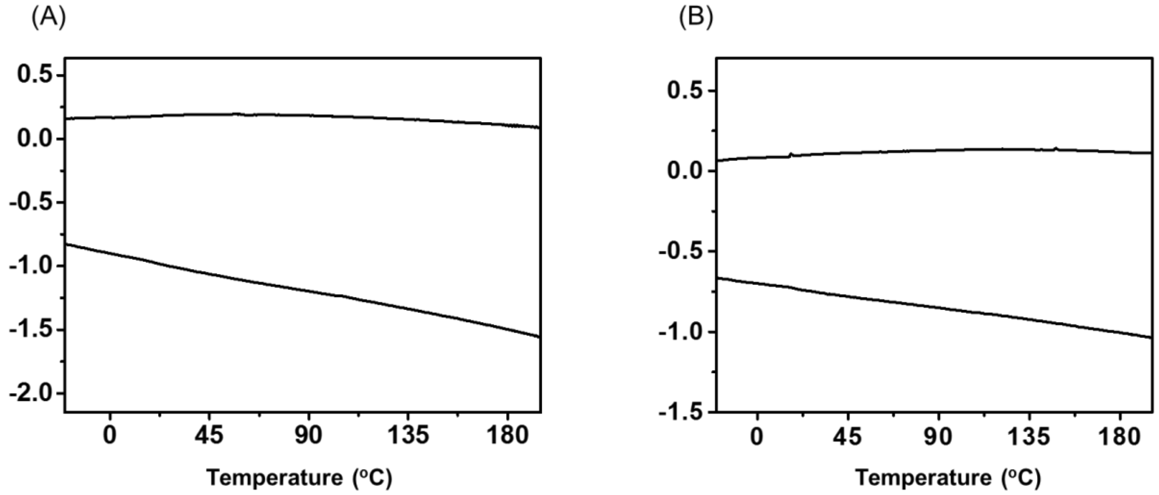

Figure S8. DSC thermograms of (**A**) BPOSS-PDI-OH and (**B**) BPOSS-PDI-C_{60}.

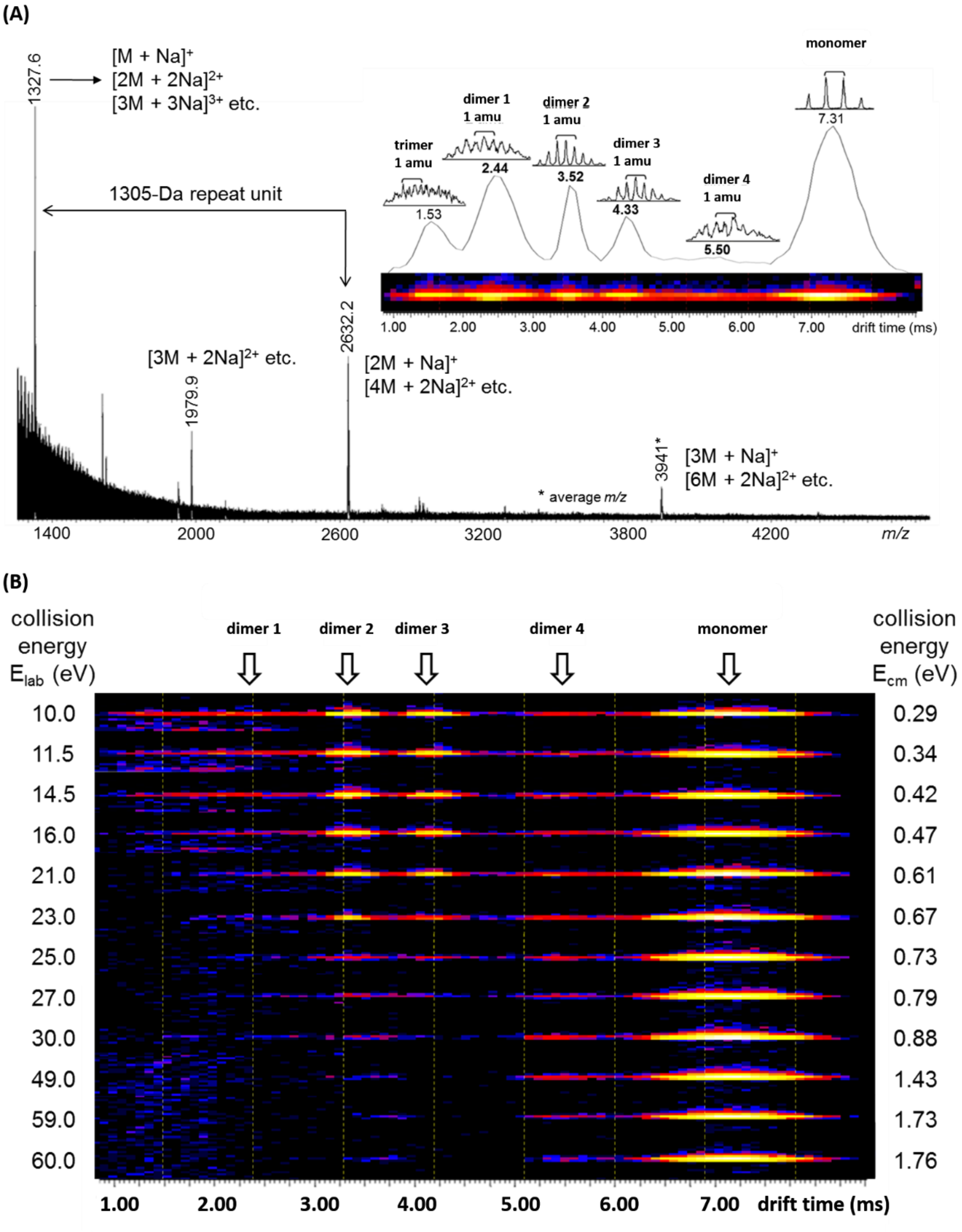

Figure S9. (**A**) ESI mass spectrum of BPOSS-PDI-OH. (**B**) Two-dimensional ESI-TWIM-MS plot of BPOSS-PDI-OH for *m*/*z* = 1327.6 showing four types of dimers and monomer at the drift time of 2.44, 3.52, 4.33, 5.50 and 7.31 ms, respectively.

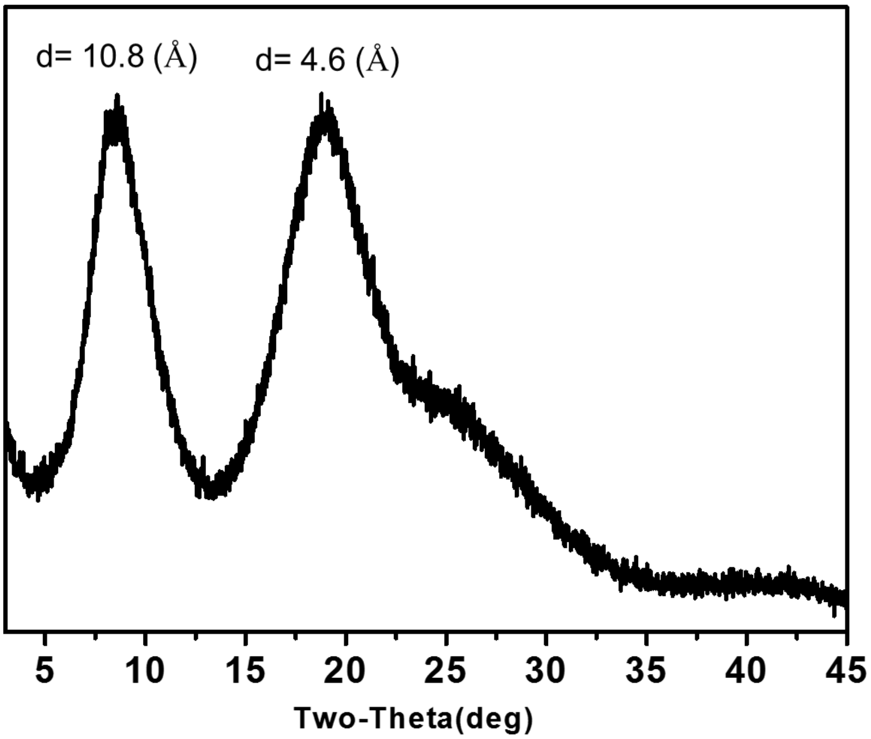

Figure S10. XRD pattern of BPOSS-PDI-C_{60}.
